# Supplementary material for: Potential environmental risk assessment of di-2-ethylhexyl phthalate emissions from a municipal solid waste landfill leachate
Source: PeerJ. 2021 Oct 1;9:e12163. doi: 10.7717/peerj.12163 (PMC8489410; doi:10.7717/peerj.12163)
Supplement: Supplemental Information 1 [file peerj-09-12163-s001.docx]

**Supplemental Data**

**Analytical methods: GC ECD method and GC MS method**

Table S1. Validation parameters of the calibration curve.

| Method | Retention time [min] | Calibration range | Calibration equation | R^2^ | LOQ |
| --- | --- | --- | --- | --- | --- |
| **GC ECD** | 19.734 | 1 – 50 µg/ml | y=31580x | 0.996 | 1.0 µg/L |
| **GC MS** | 11.211 | 0.5 - 10 µg/ml | y=1.778x | 0.99 | 1.3 µg/L |

Table S2. Validation parameters of the analytical method (RSD and recovery)

| Method |  | RSD | Recovery | RSD | Recovery |
| --- | --- | --- | --- | --- | --- |
| GC ECD |  | Spiked samples at 30 µg/L | | Spiked samples at 1 µg/L | |
|  |  | 9.24-24.00% | 82-99% | 14.56-25.20% | 54-82% |
| GC MS |  | Spiked samples at 10 µg/L | | Spiked samples at 4 µg/L | |
|  |  | 3-17% | 90-124% | 4-13% | 90-124% |
