# Supplementary material for: Potential environmental risk assessment of di-2-ethylhexyl phthalate emissions from a municipal solid waste landfill leachate
Source: PeerJ. 2021 Oct 1;9:e12163. doi: 10.7717/peerj.12163 (PMC8489410; doi:10.7717/peerj.12163)
Supplement: Supplemental Information 2 [file peerj-09-12163-s002.docx]

**Supplemental Data S2**

**IDENTIFICATION OF THE SUBSTANCE [DEHP]**

General name DEHP S

CAS-No 117-81-7 S

EC-notification no. D

EINECS no. D

Molecular weight 390.6 [g.mol-1] S

**PHYSICO-CHEMICAL PROPERTIES[DEHP]**

Melting point -55 [oC] S

Boiling point 230 [oC] S

Vapour pressure at test temperature 3.4E-05 [Pa] S

Temperature at which vapour pressure was measured 20 [oC] S

Vapour pressure at 25 [oC] 4.8E-05 [Pa] O

Water solubility at test temperature 3 [ug.l-1] S

Temperature at which solubility was measured 20 [oC] S

Water solubility at 25 [oC] 3.21 [ug.l-1] O

Octanol-water partition coefficient 7 [log10] S

Henry's law constant at 25 [oC] 5.84 [Pa.m3.mol-1] O

**ENVIRONMENT-EXPOSURE**

**RELEASE ESTIMATION**

Tonnage of substance in Europe 0 [tonnes.yr-1] O

Regional production volume of substance 0 [kg.yr-1] O

Industry category 11 Polymers industry S

Use category 47 Softeners S

**ENVIRONMENT-EXPOSURE**

**RELEASE ESTIMATION**

**[DEHP]**

Extra details on use category Polymer processing S

Extra details on use category Thermoplastics: plasticizers S

Fraction of tonnage for application 1 [-] D

**ENVIRONMENT-EXPOSURE**

**RELEASE ESTIMATION**

**[WASTE TREATMENT]**

Use specific emission scenario No D

Emission tables No applicable emission tables S

Emission scenario no special scenario selected/available S

Fraction of tonnage released to air 0 [-] O

Fraction of tonnage released to wastewater 0.967 [-] S

Fraction of tonnage released to surface water 0.033 [-] S

Fraction of tonnage released to industrial soil 0 [-] O

Fraction of tonnage released to agricultural soil 0 [-] S

Fraction of the main local source 0 [-] O

Number of emission days per year 300 [-] S

Local emission to air during episode 0 [kg.d-1] O

Local emission to wastewater during episode 0 [kg.d-1] O

Intermittent release Yes S

**ENVIRONMENT-EXPOSURE**

**RELEASE ESTIMATION**

**TOTAL REGIONAL EMISSIONS TO COMPARTMENTS[DEHP]**

Total regional emission to air 0 [kg.d-1] O

Total regional emission to wastewater 0.0304 [kg.d-1] O

Total regional emission to surface water 2.11E-03 [kg.d-1] O

Total regional emission to industrial soil 0 [kg.d-1] O

Total regional emission to agricultural soil 0 [kg.d-1] O

**ENVIRONMENT-EXPOSURE**

**PARTITION COEFFICIENTS**

**[DEHP]**

Organic carbon-water partition coefficient 1.65E+05 [l.kg-1] S

**ENVIRONMENT-EXPOSURE**

**PARTITION COEFFICIENTS**

Environmental temperature 7.6 [oC] S

**ENVIRONMENT-EXPOSURE**

**DEGRADATION AND TRANSFORMATION[DEHP]**

Characterization of biodegradability Readily biodegradable S

Degradation calculation method in STP First order, standard OECD/EU tests S

Rate constant for biodegradation in STP 0.029 [d] (DT50) S

Rate constant for biodegradation in surface water 15 [d] (DT50,20[oC]) S

Rate constant for biodegradation in bulk soil 3E+03 [d] (DT50,20[oC]) S

Rate constant for biodegradation in aerated sediment 3E+04 [d] (DT50,20[oC]) S

Rate constant for hydrolysis in surface water 1E+06 [d] (DT50,20[oC]) S

Rate constant for photolysis in surface water 1E+06 [d] (DT50) S

**ENVIRONMENT-EXPOSURE**

**SEWAGE TREATMENT**

**DEFAULT VALUES FOR SEWAGE TREATMENT**

Temperature correction for STP degradation No S

**ENVIRONMENT-EXPOSURE**

**SEWAGE TREATMENT**

**LOCAL STP[DEHP][WASTE TREATMENT]**

**OUTPUT**

Fraction of emission directed to air by STP 0.101 [%] O

Fraction of emission directed to water by STP 7 [%] S

Fraction of emission directed to sludge by STP 44.5 [%] S

Fraction of the emission degraded in STP 48.5 [%] S

Concentration in untreated wastewater 200.37 [ug.l-1] S

Concentration of chemical (total) in the STP-effluent 14 [ug.l-1] O

Concentration in effluent exceeds solubility Yes O

Concentration in dry sewage sludge 226 [mg.kg-1] O

PEC for micro-organisms in the STP 200 [ug.l-1] O

**ENVIRONMENT-EXPOSURE**

**SEWAGE TREATMENT**

**REGIONAL STP**

Fraction of emission directed to water 7 [%] S

Fraction of emission directed to sludge 44.5 [%] S

Fraction of the emission degraded 48.5 [%] S

**ENVIRONMENT-EXPOSURE**

**SEWAGE TREATMENT**

**CONTINENTAL STP**

Fraction of emission directed to water 7 [%] S

Fraction of emission directed to sludge 44.5 [%] S

Fraction of the emission degraded 48.5 [%] S

**ENVIRONMENT-EXPOSURE**

**DISTRIBUTION**

**LOCAL SCALE**

**DEFAULT VALUES FOR LOCAL SCALE**

Dry sludge application rate on agricultural soil 3E+03 [kg.ha-1.yr-1] S

Dry sludge application rate on grassland 0 [kg.ha-1.yr-1] S

**ENVIRONMENT-EXPOSURE**

**DISTRIBUTION**

**LOCAL SCALE**

**[DEHP][WASTE TREATMENT]**

Concentration in air during emission episode 0 [mg.m-3] O

Annual average concentration in air, 100 m from point source 0 [mg.m-3] O

Concentration in surface water during emission episode (dissolved) 1.12 [ug.l-1] O

Annual average concentration in surface water (dissolved) 9.24E-04 [mg.l-1] O

Local PEC in surface water during emission episode (dissolved) 1.12 [ug.l-1] O

Annual average local PEC in surface water (dissolved) 0.924 [ug.l-1] O

Local PEC in fresh-water sediment during emission episode 4.03 [mg.kgwwt-1] O

Concentration in seawater during emission episode (dissolved) 1.12E-04 [mg.l-1] O

Annual average concentration in seawater (dissolved) 9.24E-05 [mg.l-1] O

Local PEC in seawater during emission episode (dissolved) 0.112 [ug.l-1] O

Annual average local PEC in seawater (dissolved) 0.0924 [ug.l-1] O

Local PEC in marine sediment during emission episode 0.403 [mg.kgwwt-1] O

Local PEC in agric. soil (total) averaged over 30 days 1.57 [mg.kgwwt-1] O

Local PEC in agric. soil (total) averaged over 180 days 1.55 [mg.kgwwt-1] O

Local PEC in grassland (total) averaged over 180 days 1.18E-06 [mg.kgwwt-1] O

Local PEC in groundwater under agricultural soil 0.532 [ug.l-1] O

**ENVIRONMENT-EXPOSURE**

**DISTRIBUTION**

**REGIONAL AND CONTINENTAL SCALE**

**DEFAULT VALUES REGIONAL AND CONTINENTAL SCALES**

Area (land+rivers) of regional system 3.5E+04 [km2] S

Average precipitation, regional system 551 [mm.yr-1] S

**ENVIRONMENT-EXPOSURE**

**DISTRIBUTION**

**REGIONAL AND CONTINENTAL SCALE**

**CONTINENTAL[DEHP]**

Continental PEC in surface water (dissolved) 6.53E-13 [mg.l-1] O

Continental PEC in seawater (dissolved) 1.22E-13 [mg.l-1] O

Continental PEC in air (total) 4.27E-13 [mg.m-3] O

Continental PEC in agricultural soil (total) 3.14E-09 [mg.kgwwt-1] O

Continental PEC in pore water of agricultural soils 1.08E-12 [mg.l-1] O

Continental PEC in natural soil (total) 7.33E-09 [mg.kgwwt-1] O

Continental PEC in industrial soil (total) 7.33E-09 [mg.kgwwt-1] O

Continental PEC in sediment (total) 4.67E-09 [mg.kgwwt-1] O

Continental PEC in seawater sediment (total) 8.65E-10 [mg.kgwwt-1] O

**ENVIRONMENT-EXPOSURE**

**DISTRIBUTION**

**REGIONAL AND CONTINENTAL SCALE**

**REGIONAL[DEHP]**

Regional PEC in surface water (dissolved) 1.12E-05 [ug.l-1] O

Regional PEC in seawater (dissolved) 1.06E-06 [ug.l-1] O

Regional PEC in air (total) 6.11E-14 [mg.l-1] O

Regional PEC in agricultural soil (total) 1.67E-05 [mg.kgwwt-1] O

Regional PEC in pore water of agricultural soils 5.74E-06 [ug.l-1] O

Regional PEC in natural soil (total) 1.18E-06 [mg.kgwwt-1] O

Regional PEC in industrial soil (total) 1.18E-03 [ug.kgwwt-1] O

Regional PEC in sediment (total) 8.03E-05 [mg.kgwwt-1] O

Regional PEC in seawater sediment (total) 7.53E-06 [mg.kgwwt-1] O

**ENVIRONMENT-EXPOSURE**

**BIOCONCENTRATION[DEHP]**

Bioconcentration factor for earthworms 1.2E+05 [l.kgwwt-1] O

Bioconcentration factor for fish 840 [l.kgwwt-1] S

Biomagnification factor in fish 0.03 [-] S

**ENVIRONMENT-EXPOSURE**

**SECONDARY POISONING[DEHP][WASTE TREATMENT]**

Concentration in fish for secondary poisoning (freshwater) 0.0116 [mg.kgwwt-1] O

Concentration in fish for secondary poisoning (marine) 1.16E-03 [mg.kgwwt-1] O

Concentration in fish-eating marine top-predators 2.33E-04 [mg.kgwwt-1] O

Concentration in earthworms from agricultural soil 28.7 [mg.kg-1] O

**ENVIRONMENT - EFFECTS**

**MICRO-ORGANISMS[DEHP]**

Test system Inhibition control in base-set tests S

EC50 for micro-organisms in a STP ?? [mg.l-1] D

EC10 for micro-organisms in a STP ?? [mg.l-1] D

NOEC for micro-organisms in a STP 2.007E+03 [mg.l-1] S

PNEC for micro-organisms in a STP 201 [mg.l-1] O

Assessment factor applied in extrapolation to PNEC micro 10 [-] S

**ENVIRONMENT - EFFECTS**

**FRESH_WATER ORGANISMS[DEHP]**

LC50 for fish ?? [ug.l-1] D

L(E)C50 for Daphnia ?? [ug.l-1] D

EC50 for algae ?? [ug.l-1] D

LC50 for additional taxonomic group ?? [ug.l-1] D

NOEC for fish ?? [ug.l-1] D

NOEC for Daphnia ?? [ug.l-1] D

NOEC for algae ?? [mg.l-1] D

NOEC for additional taxonomic group ?? [ug.l-1] D

PNEC for aquatic organisms 0.07 [ug.l-1] O

PNEC for aquatic organisms, intermittent releases 0.07 [ug.l-1] O

Assessment factor applied in extrapolation to PNEC Aqua 2.857 [-] S

Assessment factor applied in extrapolation to PNEC Aqua 2.857 [-] S

PNEC for aquatic organisms with statistical method 0.07 [ug.l-1] S

**ENVIRONMENT - EFFECTS**

**MARINE ORGANISMS[DEHP]**

LC50 for fish (marine) ?? [mg.l-1] D

L(E)C50 for crustaceans (marine) ?? [mg.l-1] D

EC50 for algae (marine) ?? [mg.l-1] D

LC50 for additional taxonomic group (marine) ?? [mg.l-1] D

NOEC for fish (marine) ?? [mg.l-1] D

NOEC for crustaceans (marine) ?? [mg.l-1] D

NOEC for algae (marine) ?? [mg.l-1] D

NOEC for additional taxonomic group (marine) ?? [mg.l-1] D

PNEC for marine organisms ?? [mg.l-1] O

**ENVIRONMENT - EFFECTS**

**FRESH-WATER SEDIMENT ORGANISMS[DEHP]**

LC50 for fresh-water sediment organism ?? [mg.kgwwt-1] D

EC10 for fresh-water sediment organism ?? [mg.kgwwt-1] D

EC10 for fresh-water sediment organism ?? [mg.kgwwt-1] D

EC10 for fresh-water sediment organism ?? [mg.kgwwt-1] D

NOEC for fresh-water sediment organism 1000 [mg.kgdwt-1] S

NOEC for fresh-water sediment organism ?? [mg.kgwwt-1] D

NOEC for fresh-water sediment organism ?? [mg.kgwwt-1] D

PNEC for fresh-water sediment, normalised to 5% o.c. (regional) 100 [mg.kgdwt-1] O

**ENVIRONMENT - EFFECTS**

**MARINE SEDIMENT ORGANISMS[DEHP]**

LC50 for marine sediment organism ?? [mg.kgwwt-1] D

EC10 for marine sediment organism ?? [mg.kgwwt-1] D

EC10 for marine sediment organism ?? [mg.kgwwt-1] D

EC10 for marine sediment organism ?? [mg.kgwwt-1] D

NOEC for marine sediment organism ?? [mg.kgwwt-1] D

NOEC for marine sediment organism ?? [mg.kgwwt-1] D

NOEC for marine sediment organism ?? [mg.kgwwt-1] D

PNEC for marine sediment, normalised to 5% o.c. (regional) 0.217 [mg.kgwwt-1] O

**ENVIRONMENT - EFFECTS**

**TERRESTRIAL ORGANISMS[DEHP]**

LC50 for plants ?? [mg.kgwwt-1] D

LC50 for earthworms ?? [mg.kgwwt-1] D

EC50 for microorganisms ?? [mg.kgwwt-1] D

LC50 for other terrestrial species ?? [mg.kgwwt-1] D

NOEC for plants ?? [mg.kgwwt-1] D

NOEC for earthworms ?? [mg.kgwwt-1] D

NOEC for microorganisms 300 [mg.kgdwt-1] S

NOEC for additional taxonomic group ?? [mg.kgwwt-1] D

NOEC for additional taxonomic group ?? [mg.kgwwt-1] D

PNEC for terrestrial organisms 13 [mg.kgdwt-1] O

Equilibrium partitioning used for PNEC in soil? No S

Assessment factor applied in extrapolation to PNEC Terr 23 [-] S

PNEC for terrestrial organisms with statistical method 13 [mg.kgdwt-1] S

**ENVIRONMENT - EFFECTS**

**BIRDS AND MAMMALS[DEHP]**

NOEC via food (birds) 1.7E+03 [mg.kg-1] S

Duration of (sub-)chronic oral test 28 days D

NOEC via food for secondary poisoning 33.3 [mg.kg-1] S

PNEC for secondary poisoning of birds and mammals 3.33 [mg.kg-1] O

Assessment factor applied in extrapolation to PNEC oral 10 [-] S

**ENVIRONMENT - RISK CHARACTERIZATION**

**LOCAL[DEHP][WASTE TREATMENT]**

RCR for the local fresh-water compartment 16.1 [-] O

RCR for the local fresh-water compartment, statistical method 16.1 [-] O

RCR for the local marine compartment ?? [-] O

RCR for the local marine compartment, statistical method ?? [-] O

RCR for the local fresh-water sediment compartment 0.0928 [-] O

RCR for the local marine sediment compartment 0.928 [-] O

RCR for the local soil compartment 0.136 [-] O

RCR for the local soil compartment, statistical method 0.137 [-] O

RCR for the sewage treatment plant 9.98E-04 [-] O

RCR for fish-eating birds and mammals (fresh-water) 3.5E-03 [-] O

RCR for fish-eating birds and mammals (marine) 3.5E-04 [-] O

RCR for top predators (marine) 6.99E-05 [-] O

RCR for worm-eating birds and mammals 8.63 [-] O

**ENVIRONMENT - RISK CHARACTERIZATION**

**REGIONAL**

RCR for the regional fresh-water compartment 1.61E-04 [-] O

RCR for the regional fresh-water compartment, statistical method 1.61E-04 [-] O

RCR for the regional marine compartment ?? [-] O

RCR for the regional marine compartment, statistical method ?? [-] O

RCR for the regional fresh-water sediment compartment 3.69E-06 [-] O

RCR for the regional marine sediment compartment 3.46E-05 [-] O

RCR for the regional soil compartment 1.45E-06 [-] O

RCR for the regional soil compartment, statistical method 1.46E-06 [-] O
