# Supplementary material for: Potential environmental risk assessment of di-2-ethylhexyl phthalate emissions from a municipal solid waste landfill leachate
Source: PeerJ. 2021 Oct 1;9:e12163. doi: 10.7717/peerj.12163 (PMC8489410; doi:10.7717/peerj.12163)
Supplement: Supplemental Information 4 [file peerj-09-12163-s004.docx]

**Table S3.** DEHP concentrations in groundwater (μg/ L)

| Year of sampling | Season of sampling | DEHP concentration in groundwater (μg/L) | |
| --- | --- | --- | --- |
|  |  | upstream | downstream |
| 2014 | Summer | - | - |
|  |  | - | - |
|  |  | - | - |
|  | Autumn | - | - |
|  |  | - | - |
|  |  | - | - |
|  | Winter | - | - |
|  |  | - | - |
|  |  | - | - |
| 2015 | Summer | <LOQ | <LOQ |
|  |  | <LOQ | <LOQ |
|  |  | <LOQ | <LOQ |
|  | Autumn | <LOQ | - |
|  |  | <LOQ | - |
|  |  | <LOQ | - |
|  | Winter | <LOQ | <LOQ |
|  |  | <LOQ | <LOQ |
|  |  | <LOQ | <LOQ |
| 2016 | Spring | - | - |
|  |  | - | - |
|  |  | - | - |
|  | Summer | - | - |
|  |  | - | - |
|  |  | - | - |
|  | Autumn | 1.5 | - |
|  |  | <LOQ | - |
|  |  | <LOQ | - |

**Table S4.** DEHP concentrations in raw MSW landfill leachate (μg/L)

| Year of sampling | Season of sampling | DEHP concentration in leachate (μg/L) |
| --- | --- | --- |
|  |  |  |
| 2014 | Summer | 18.5 |
|  |  | 22.3 |
|  |  | 17.8 |
|  | Autumn | 256.7 |
|  |  | 394.4 |
|  |  | - |
|  | Winter | 167.0 |
|  |  | 184.3 |
|  |  | 22.5 |
| 2015 | Summer | 64.9 |
|  |  | 65.4 |
|  |  | 73.9 |
|  | Autumn | 30.2 |
|  |  | 58.1 |
|  |  | 9.1 |
|  | Winter | 52.6 |
|  |  | 44.6 |
|  |  | 34.4 |
| 2016 | Spring | 43.1 |
|  |  | 36.0 |
|  |  | 32.4 |
|  | Summer | 7.5 |
|  |  | 12.0 |
|  |  | 7.1 |
|  | Autumn | <LOQ |
|  |  | <LOQ |
|  |  | <LOQ |

**Table S5.** Observed precipitation at Pruszków weather station [mm]

|  | **Year** | | |
| --- | --- | --- | --- |
| **Month** | **2014** | **2015** | **2016** |
| January | 60.6 | 42.9 | 21.4 |
| February | 19.0 | 11.0 | 70.5 |
| March | 35.8 | 33.6 | 31.5 |
| April | 48.8 | 31.2 | 27.7 |
| May | 87.7 | 39.5 | 30.1 |
| June | 69.0 | 20.1 | 115.3 |
| July | 108.4 | 70.2 | 61.4 |
| August | 66.9 | 7.2 | 47.2 |
| September | 20.5 | 36.3 | 31.8 |
| October | 4.7 | 45.1 | 118.5 |
| November | 24.1 | 54.8 | 39.4 |
| December | 76.9 | 15.7 | 72.8 |
| **Total** | **622.4** | **407.6** | **667.6** |
